# The juvenile alopecia mutation (*jal*) maps to mouse Chromosome 2, and is an allele of GATA binding protein 3 (*Gata3*)

**DOI:** 10.1186/1471-2156-14-40

**Published:** 2013-05-09

**Authors:** Francisco Ramirez, Aaron M Feliciano, Elisabeth B Adkins, Kevin M Child, Legairre A Radden II, Alexis Salas, Nelson Vila-Santana, José M Horák, Samantha R Hughes, Damek V Spacek, Thomas R King

**Affiliations:** 1 Biomolecular Sciences, Central Connecticut State University, 1615 Stanley Street, New Britain, CT 06053, USA

**Keywords:** Mouse model, Focal alopecia, Positional candidate approach, *Il2ra*, *Gata3*, Complementation testing

## Abstract

**Background:**

Mice homozygous for the juvenile alopecia mutation (*jal*) display patches of hair loss that appear as soon as hair develops in the neonatal period and persist throughout life. Although a report initially describing this mouse variant suggested that *jal* maps to mouse Chromosome 13, our preliminary mapping analysis did not support that claim.

**Results:**

To map *jal* to a particular mouse chromosome, we produced a 103-member intraspecific backcross panel that segregated for *jal,* and typed it for 93 PCR-scorable, microsatellite markers that are located throughout the mouse genome. Only markers from the centromeric tip of Chromosome 2 failed to segregate independently from *jal*, suggesting that *jal* resides in that region. To more precisely define *jal*’s location, we characterized a second, 374-member backcross panel for the inheritance of five microsatellite markers from proximal Chromosome 2. This analysis restricted *jal*’s position between *D2Mit359* and *D2Mit80*, an interval that includes *Il2ra* (for interleukin 2 receptor, alpha chain), a gene that is known to be associated with alopecia areata in humans. Complementation testing with an engineered null allele of *Il2ra*, however, showed that *jal* is a mutation in a distinct gene. To further refine the location of *jal*, the 374-member panel was typed for a set of four single-nucleotide markers located between *D2Mit359* and *D2Mit80*, identifying a 0.55 Mb interval where *jal* must lie. This span includes ten genes—only one of which, *Gata3* (for GATA binding protein 3)—is known to be expressed in skin. Complementation testing between *jal* and a *Gata3* null allele produced doubly heterozygous, phenotypically mutant offspring.

**Conclusions:**

The results presented indicate that the *jal* mutation is a mutant allele of the *Gata3* gene on mouse Chromosome 2. We therefore recommend that the *jal* designation be changed to *Gata3*^*jal*^*,* and suggest that this mouse variant may provide an animal model for at least some forms of focal alopecia that have their primary defect in the hair follicle and lack an inflammatory component.

## Background

**Figure 1:**
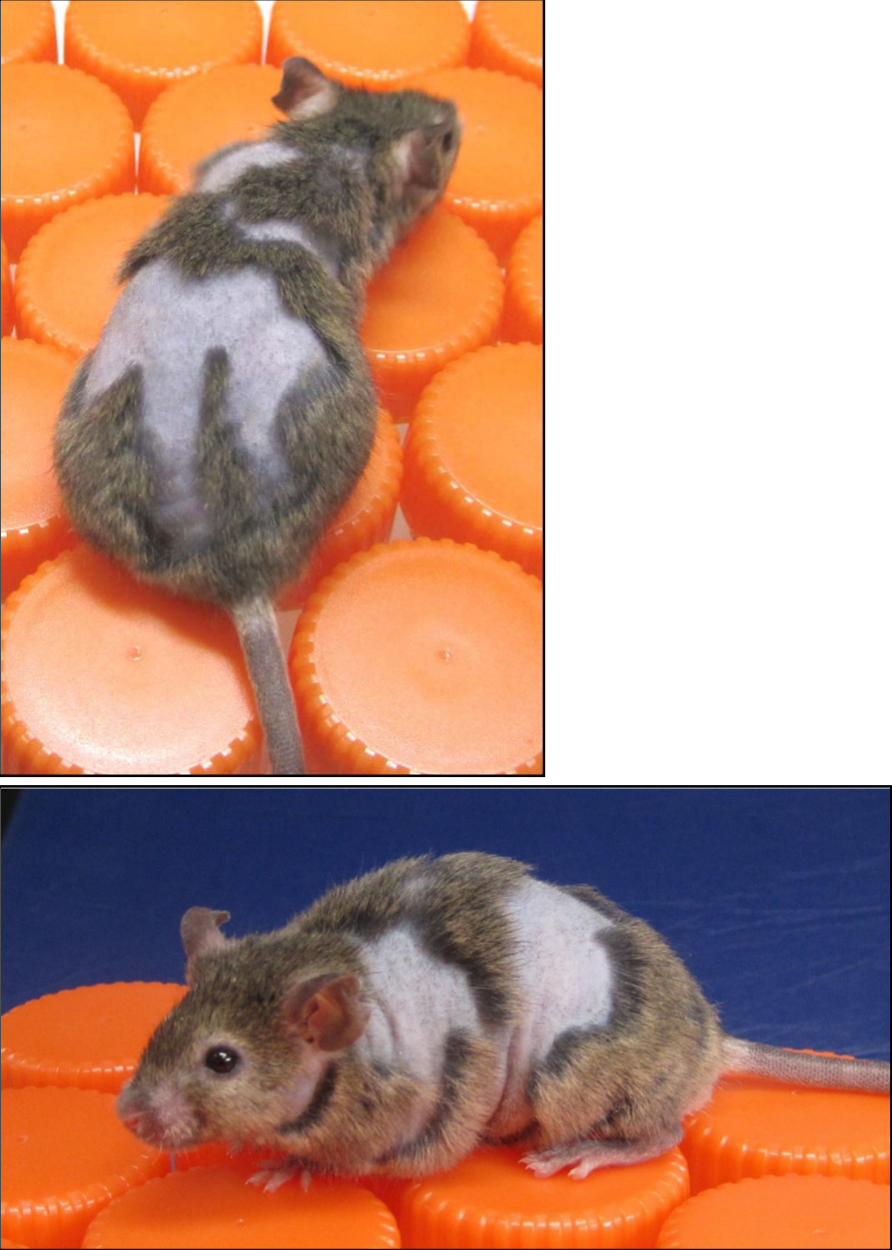
**A three-month-old C3H/HeJ-*jal*/J mouse, homozygous for *jal*.**

The initial assignment of spontaneous hair variants to particular genes can be a crucial first step in the long-term investigation into the role these genes play in the normal (and disrupted) development of the mammalian integument (for example, see refs. [1-9]). Unfortunately, several naturally-occurring hair and skin variants in mice remain out-of-the-mainstream of modern biological investigation, simply because they have not yet been assigned to a causative gene or even, in some cases, to a particular chromosome. One such variant is generated by the recessive juvenile alopecia mutation, abbreviated *jal*. This variant arose on the standard C3H/HeJ genetic background, and its origin and novel phenotype were described in a single brief paper published by McElwee *et al*. in 1999 [10]. Homozygous mice exhibit patchy hair loss (see Figure 1), wavy truncal hair, defects in hair follicles, and abnormalities in hair growth cycle regulation. Vibrissae defects are apparent at birth, and focal alopecia is evident as soon as hair develops in the neonatal period. Although McElwee and coworkers suggested that *jal* is located on mouse Chromosome (Chr) 13 [10], our preliminary backcross analysis [11] clearly showed that *jal* does not map anywhere on that chromosome.

Here, we describe the completed molecular-genetic analysis of a pair of large backcross families that allowed us to locate *jal* on mouse Chr 2, and then restrict its location to a small, defined interval at the centromeric tip. In addition, we describe complementation testing between *jal* and engineered null alleles of two co-localizing candidate genes, one of which (*Gata3*, for GATA binding protein 3) we identify as the likely basis of the juvenile alopecia phenotype in mice.

## Methods

### Mice

Mice from the standard inbred strains C57BL/6 J, C3H/HeJ, A/J, as well as inbred C3H/HeJ-*jal*/J mice were obtained from The Jackson Laboratory (Bar Harbor, ME, USA). Mice homozygous for the mutant *jal* allele were most reliably identified by vibrissae defects that are first evident shortly after birth. By two weeks of age, homozygotes present with distinct patches of hair loss (most often on the dorsal surface) that persist throughout life (see Figure 1). The amount of body surface affected varies widely among homozygous individuals (from less than 5% to greater than 95% [see Additional file 1]), even within the inbred C3H/HeJ-*jal* strain. While both male and female *jal/jal* homozygotes are fertile, we have maintained the C3H/HeJ-*jal* line since 2009 by crossing heterozygous females with homozygous males, to produce segregating litters.

Mice carrying a targeted mutation in the interleukin 2 receptor, alpha chain gene (*Il2ra*^*tm1Dw*^) were also obtained from The Jackson Laboratory. The creation of the *Il2ra*^*tm1Dw*^ loss-of-function allele is described by Willerford *et al.*[12]. In brief, these investigators used homologous recombination to replace a 5.5 kb segment of the *Il2ra* gene which contains Exons 2 and 3 and encodes the interleukin 2 binding site [13] with a phosphoglycerate kinase (PGK)-neomycin resistance (*neo*) cassette. Mice carrying a targeted mutation in the GATA binding protein 3 gene (*Gata3*^*tm1Gsv*^) were kindly donated by Dr. James Douglas Engel (University of Michigan, Ann Arbor, MI, USA). The creation of the *Gata3*^*tm1Gsv*^ loss-of-function/reporter allele is described by van Doorninck *et al.*[14]. In brief, these investigators replaced 157 bp in Exon 2, including the start codon, with a nuclear localization signal (*nls*)-*lacZ* fusion cassette, followed by a PGK-hygromycin resistance (*hyg*) cassette.

All studies were in compliance with protocols approved by the Institutional Care and Use Committee (IACUC) at Central Connecticut State University (New Britain, CT, USA).

### DNA isolation and analysis

Genomic DNA was isolated from 3 mm tail tip biopsies taken from two-week-old mice, using Nucleospin kits from BD Biosciences (Palo Alto, CA, USA). The polymerase chain reaction (PCR) was performed using the Titanium PCR kit from Clontech (Palo Alto, CA, USA). Oligonucleotide primers for PCR were synthesized by Invitrogen (Carlsbad, CA, USA), based on sequence information from online sources [15,16]. In addition to standard, PCR-scorable, microsatellite markers [17], we also assayed 4 markers based on single-nucleotide polymorphisms that have been reported to differ between the A/J and C3H/HeJ strains [15,16]. These markers, designated herein as *SNP1-4*, are described in detail in Additional file 2 and Additional file 3. To distinguish between *Il2ra*^*tm1Dw*^ carriers and wild type mice, we used the 4-primer PCR assay recommended by the mouse supplier (The Jackson Laboratory). Two of these primers (5′CTGTGTCTGTATGACCCACC 3′, and 5′ CAGGAGTTTCCTAAGCAACG 3′) correspond to Exon 2 of *Il2ra*, which in the mutant has been replaced with a PGK-*neo* cassette, and yield a 280 bp amplimer with wild type DNA templates. The other two primers (5′ CTTGGGTGGAGAGGCTATTC 3′, and 5′AGGTGAGATGACAGGAGATC 3′) correspond to the *neo* gene, and direct the amplification of a 280 bp amplimer from mutant DNA templates. To distinguish between *Gata3*^*tm1Gsv*^ carriers and wild type mice, we used a 3-primer PCR assay of our own design. For this test, one primer-pair (forward primer, 5′ CCCTAAACCCTCCTTTTTGC 3′, and reverse primer 5′ GATACCTCTGCACCGTAGCC 3′) flanked the site of the engineered disruption in Exon 2, and produced a 399 bp amplimer with wild type templates; that forward primer and second reverse primer (5′ GTTTTCCCAGTCACGACGTT 3′), based on sequences within in *lacZ*, yielded a 320 bp amplimer that is specific to the *Gata3*^*tm1Gsv*^ allele.

PCR products plus 2 ul loading buffer (bromophenol blue in 20% Tris-buffered sucrose) were electrophoresed through 3.25% NuSeive 3:1 agarose gels (Lonza, Rockland, ME, USA). Gels were stained with ethidium bromide (0.5 ug/mL) and photographed under ultraviolet light. For sequence analysis, about 1.5 ug of individual PCR amplimers were concentrated into a 30 ul volume using QIAquick PCR Purification kits (Qiagen, Valencia, CA, USA). Purified amplimers were shipped to SeqWright, Inc. (Houston, TX, USA) for primer-extension analysis.

### mRNA analysis

Total RNA was isolated from skin and thymus samples taken from 1-month-old mutant and wild type mice mice using the Nucleospin^®^ RNA L kit by Macherey-Nagel (Easton, PA, USA). cDNA was generated using the SMARTer™ RACE cDNA amplification kit (Clontech Laboratories). To amplify *Gata3*-specific cDNA, primer pairs that flanked exon junction boundaries were used in “step-down” PCR reactions. The products of this initial reaction were diluted 1:10 in Tricine-KOH buffer (10 mM, pH 8.5) plus 1 mM EDTA, and were amplified again in standard PCR reactions using the same or nested primer pairs. Second-round amplimers were purified (as described above) and shipped to SeqWright, Inc., for primer-extension sequencing.

## Results

### Mapping *jal* to a mouse chromosome

To determine if *jal* might be carried on the mouse X chromosome, we conducted reciprocal crosses of homozygous mutant mice with wild type mice from the C57BL/6 J strain. Since the F_1_ progeny of both genders were phenotypically wild type [see Additional file 4], we confirm that the *jal* mutation is recessive, and conclude that it must reside in an autosomal portion of the genome.

**Figure 2:**
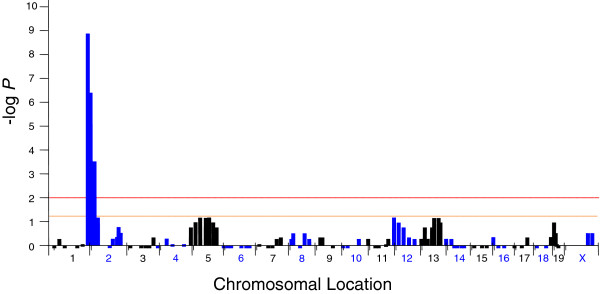
**Inheritance of *jal *and 93 microsatellite markers, tested for goodness-of-fit with an independent-assortment model. **Each microsatellite marker tested is represented by a single bar positioned on the horizontal axis to show its approximate location in the mouse genome. Markers from odd chromosomes are in black, those from even chromosomes are in blue. Results are plotted as negative log-transformed *P* values calculated by the chi-squared method (with 1 degree of freedom). Bars descend below the baseline for those markers where more recombinant types (*i.e.*, *jal *inherited from the F_1 _mother together with a C57BL/6-derived marker allele, or *jal*^*+ *^inherited with a C3H/HeJ-derived marker) than parental types (*jal* inherited from the F_1 _mother together with a C3H/HeJ-derived marker allele, or *jal*^*+ *^inherited with a C57BL/6-derived marker) were observed in a set of 44 family members initially typed. Additional mice (up to all 103 in the backcross panel) were typed for markers that showed a surplus of parental types such that goodness-of-fit testing with the expected 1:1 ratio gave *P *< 0.1. Only markers from proximal Chr 2 showed a significant (above the orange line, where *P *< 0.05) or highly significant (above the red line, where *P *< 0.01) excess of parental types, indicative of linkage with *jal*.

To determine an autosomal location for the *jal* mutation, we crossed (C57BL/6 J × C3H/HeJ-*jal*)F_1_*jal/+* females back to their *jal*/*jal* sire. This cross produced 43 mutants and 60 wild type progeny, not significantly different from the 1 mutant : 1 wild type ratio expected for a testcross (*χ*^2^ = 2.81; *P* > 0.09). DNA samples isolated from these 103 backcross (N_2_) progeny were analyzed for 93 PCR-scorable microsatellite markers from throughout the mouse genome, including two from the pseudoautosomal region on the X and Y chromosomes. The average spacing of these markers was 16 cM, with the largest gap being a 31 cM interval on Chr 4. Among the markers tested, only those from the centromeric portion of Chr 2 showed an inheritance pattern significantly different from the 1 parental : 1 recombinant ratio predicted if the marker and *jal* were independently assorted (see Figure 2). The largest deviation (82 parental and 21 recombinant types; *χ*^2^ = 36.13; *P* < 1.85 × 10^-9^) was observed for marker *D2Mit1*, which is located 2.23 cM from the centromeric end of Chr 2 [15].

### Meiotic fine-mapping

**Figure 3:**
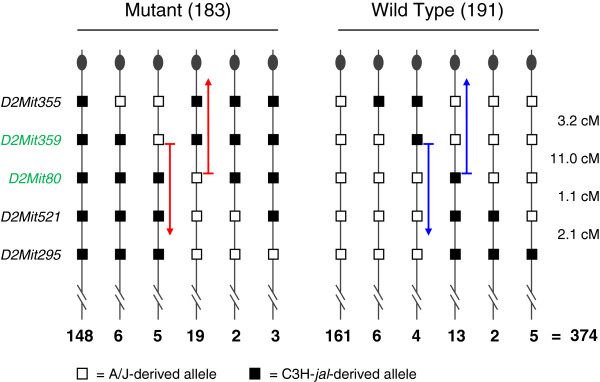
**Segregation of *jal *and five microsatellite markers on proximal Chr 2 among 374 backcross mice.** The five markers typed are shown to the left of the diagram. The haplotype transmitted by the heterozygous F_1 _dam is depicted. Open boxes indicate A/J-derived alleles; solid boxes indicate C3H/HeJ-derived alleles. The centromere is indicated by a knob at the top of each haplotype. The number of progeny inheriting each haplotype is shown below it. Genetic distances are shown to the right. The red arrows indicate that in these recombinant mutants, the mutant *jal *allele must be located below *D2Mit359*, but above *D2Mit80*. The blue arrows similarly indicate that in these recombinant wild type mice, the normal *jal*^*+ *^allele must be located below *D2Mit359*, but above *D2Mit80*.

To more precisely locate *jal* on proximal Chr 2, we bred (A/J × C3H/HeJ-*jal*/J)F_1_, *jal/+* females back to C3H/HeJ-*jal/jal* males, since this strain combination offered more microsatellite and single nucleotide polymorphisms (SNPs) than the C57BL/6 J and C3H/HeJ strain combination. These N_2_ mice were typed for *jal* and six microsatellite markers on proximal Chr 2, as summarized in Figure 3. The 374 progeny from this backcross generation fit well with the expected 1 wild type : 1 mutant ratio expected for a testcross (*χ*^2^ = 0.17; *P* > 0.67), so mutants appear to be equally viable as their wild type, heterozygous littermates. Segregation of markers among this large N_2_ family indicates that *jal* is located between *D2Mit359* and *D2Mit80*, a span of about 11 cM that contains some 11.66 Mb of DNA [16].

### Complementation testing between *jal* and a targeted mutation in *Il2ra*

A recent genome-wide association study for alopecia areata (AA, OMIM #104000) in humans has implicated several genes, including *IL-2RA* (for interleukin 2 receptor, alpha chain) in the development of disfiguring hair loss [18]. Because AA appears similar in at least some ways to the mutant *jal/jal* phenotype in mice [10], and because *IL-2RA* is located on human Chr 10p15.1—a region that is orthologous with the *D2Mit359* and *D2Mit80* interval on Chr 2 in mouse—we decided to test *jal* for complementation with the recessive *Il2ra*^*tm1Dw*^ loss-of-function mouse mutation [12]. Because mice homozygous for the targeted mutation show poor survival, we crossed *Il2ra*^*tm1Dw*^*/+* heterozygous females with *jal/jal* males. If *jal* were a defect in *Il2ra*, then the mice that inherit *jal* and *Il2ra*^*tm1Dw*^ could express no wild-type gene product, and would therefore be expected to show some mutant phenotype, perhaps as mild as defective vibrissae (as displayed by all *jal/jal* mutants) or perhaps as severe as the slower growth and progressive wasting (cachexia) seen in mice homozygous for *Il2ra*^*tm1Dw*^[19]. Alternatively, if *jal* and *Il2ra* are distinct genes, then all of the progeny would be phenotypically normal (since both mutations are recessive).

This cross yielded 19 offspring that were typed by PCR for the *Il2ra*^*tm1Dw*^ targeted disruption [Additional file 5] and observed for 30 weeks. DNA typing identified 11 *Il2ra*^*tm1Dw*^ carriers (5 females and 6 males) and 8 mice without the targeted disruption (7 females and 1 male), not significantly different from the 1:1 ratio expected for a test cross (*χ*^2^ = 0.47; *P* = 0.49). All of these mice (*Il2ra*^*tm1Dw*^ carriers and noncarriers) displayed normal vibrissae and body hair. Furthermore, *Il2ra*^*tm1Dw*^ carriers and noncarriers showed indistinguishable growth rates (over a period of 30 weeks), with no signs of the cachexia seen in *Il2ra*^*tm1Dw*^*/Il2ra*^*tm1Dw*^ controls [Additional file 6]. These data suggest that *jal* is not an allele of *Il2ra*.

### Refinement of the meiotic map for *jal*

**Figure 4:**
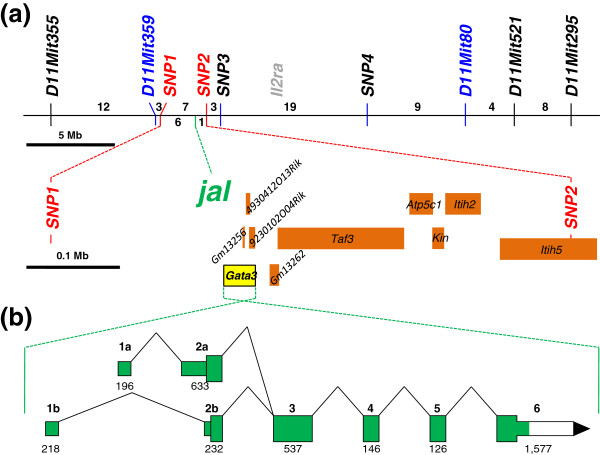
**Physical maps of the *jal *region on mouse Chr 2. **(**a**) Molecular markers and genes on mouse Chr 2 that are linked with *jal*. Segregation data from the 374-member backcross panel shown in Figure 3 placed *jal *between microsatellite markers *D2Mit359 *and *D2Mit80 *(shown in blue), an interval that also includes *Il2ra *(shown in gray). Single-nucleotide polymorphisms (*SNP1-4*, see Additional file 2 and Additional file 3) were used to more precisely locate crossovers among the 41 mice recombinant in this interval. The number of crossovers located between the various pairs of adjacent markers are shown on the chromosome map, which is drawn to the 5 Mb scale shown. Seven recombinants located *jal* between *SNP1 *and *SNP2 *(shown in red). The region between *SNP1 *and *SNP2 *is expanded below the chromosome map (drawn to the 0.1 MB scale bar shown), to show the locations of the 10 candidate genes (represented by orange boxes) that populate this span. Of these ten genes, only one, *Gata3 *(shown in yellow), is known to be expressed in skin. (**b**) The *Gata3 *gene is expanded to show the arrangement of exons, where taller boxes are coding regions and shorter boxes are the 5′ or 3′ untranslated regions. *Gata3* is transcribed from the reverse strand, but is drawn here so that the six exons are shown in ascending numerical order. The length of each exon (in bp) is shown below the corresponding box. The portions of exons shaded green have been sequenced in C3H/HeJ and C3H/HeJ-*jal*/J DNA, but no differences were found.

The 41 mice from the (A/J x C3H/HeJ-*jal*/J)F_1_ × C3H/HeJ-*jal*/J backcross that were recombinant in the *D2Mit359* and *D2Mit80* interval were next typed for four, single-nucleotide polymorphisms designated *SNP1*, *SNP2*, *SNP3* and *SNP4* (see Additional file 2 and Additional file 3). This analysis identified six crossovers between *SNP1* and *jal*, and one crossover between *jal* and *SNP2*, placing the *jal* mutation between these two markers (see Figure 4a), a 0.55 Mb span that does not include *Il2ra*. Of the ten genes or predicted genes [16] that do map to this interval, only one—*Gata3* (for GATA binding protein 3)—is known to be expressed in skin [20,21].

### Evaluation of *Gata3* as the possible genetic basis of the *jal* mutation

To determine if *jal* could be a mutant allele of the *Gata3* gene, we imported a mouse carrying an engineered *Gata3* null allele, *Gata3*^*tm1Gsv*^[14], for complementation testing. To create litters of half experimental (doubly heterozygous) and half control offspring (carriers of the *jal* allele, only), we crossed *Gata3*^*tm1Gsv*^*/+* heterozygous females with *jal/jal* males. If *jal* is the result of a defect in *Gata3*, then the mice that inherit both *jal* and *Gata3*^*tm1Gsv*^ could express no wild-type gene product, and would therefore be expected to show defective coats and vibrissae. Alternatively, if *jal* and *Gata3* are distinct genes, then the dihybrid progeny (*jal*/+, *Gata3*^*tm1Gsv*^/+) would be phenotypically normal.

**Figure 5:**
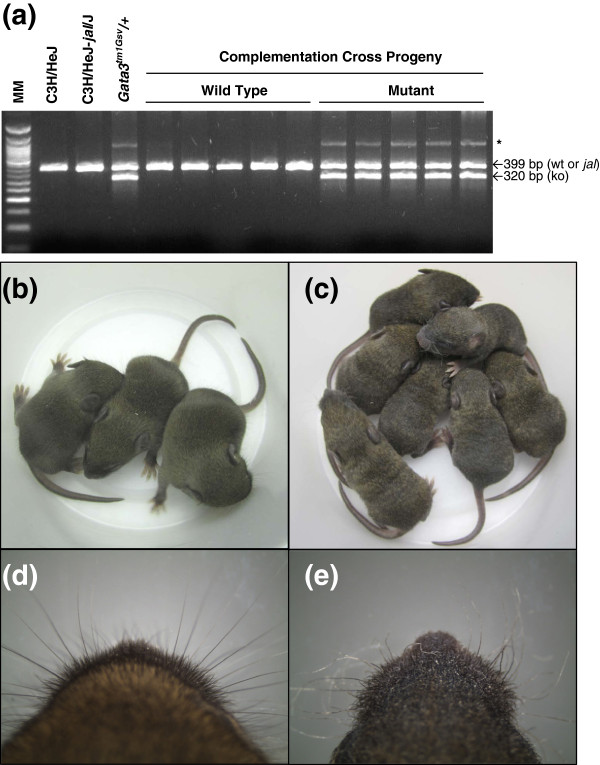
**The recessive *jal *and *Gata3***^***tm1Gsv ***^**mutations fail to complement in doubly heterozygous mice. **(**a**) Typical results of a 3-primer PCR test designed to identify *Gata3*^*tm1Gsv *^carriers. The 320 bp band and a fainter, high-molecular-weight band (marked with an asterisk) are specific to the mutant allele. The size standard shown (MM) is a 50 base pair ladder. A 10-day-old litter from a cross of *Gata3*^*tm1Gsv*^/*Gata3*^*+ *^x *jal*/*jal *included pups displaying wild type (**b**) or mutant (**c**) hair development. All phenotypically wild type pups showed the 399 bp band only, and the phenotypically mutant pups all carried the targeted mutation. The snouts of one wild type (**d**) and one mutant (**e**) pup from the same litter are enlarged to show normal *vs*. defective vibrissae formation, respectively.

This cross yielded 22 offspring that were typed by PCR for the *Gata3*^*tm1Gsv*^ targeted disruption. DNA typing identified 11 *Gata*^*tm1Gsv*^ carriers (6 females and 5 males) and 11 mice without the disruption (10 females, 1 male), as expected for a test cross (Figure 5a). All *Gata3*^*tm1Gsv*^ carriers displayed defective vibrissae and body hair (see Figure 5c and e), while those without the targeted mutation in *Gata3* appeared phenotypically normal (Figure 5b and d). Thus, *jal* and *Gata3*^*tm1Gsv*^ fail to complement, suggesting that these mutations are allelic.

All coding regions of the *Gata3* gene, plus the 5′ untranslated regions encoded by two alternative 1st exons (see Figure 4b for transcript diagram and summary) were sequenced in DNA isolated from C3H/HeJ and C3H/HeJ-*jal*/J mice. However, we found no differences in DNA sequence between these coisogenic wild type and mutant strains. In addition, using total RNA isolated from skin and from thymus, we amplified (and sequenced) only identically-spliced *Gata3* cDNA from both wild type C3H/HeJ and C3H/HeJ-*jal*/J mutant mice (see Additional file 7).

## Discussion

The results presented suggest to us that the *jal* mutation is a mutant allele of the *Gata3* gene on mouse Chr 2. We therefore recommend that the *jal* designation be changed to *Gata3*^*jal*^. While we have not yet been able to pinpoint a sequence-level change in *Gata3*^*jal*^, our analysis has mostly been limited to coding regions. We hypothesize that the *Gata3*^*jal*^ defect is likely to be a regulatory mutation (perhaps located in the promoters, introns, or 3′ untranslated region) that—in some fashion—impacts expression, processing, or degradation of the *Gata3*^*jal*^ transcript, although we find that the *Gata3*-*001* transcript appears to be normally spliced. (We found no evidence for expression of the alternative *Gata3-201* transcript in total RNA isolated from skin or thymus.) Quantitative and qualitative evaluation of *Gata3* transcripts or protein in the epidermis and hair follicles of C3H/HeJ-*jal* mice versus wild type controls could help refine this array of possibilities. This prediction (that the *Gata3*^*jal*^ defect is likely to be a regulatory mutation) does seem consistent with the variable phenotypic presentation of focal alopecia that we observe in *Gata3*^*jal*^/*Gata3*^*jal*^ mice (see Additional file 1). Since at least some patches of normal fur are seen on most if not all mutants (with some mutants showing almost entirely normal coats), we anticipate that a standard primary protein sequence (albeit improperly regulated) is likely to be encoded by the *Gata3*^*jal*^ allele.

The positional assignment of *jal* did not reveal (as with our introductory examples, refs. 1–9) an unsuspected function of *Gata3* in skin, since the study of mouse strains engineered to carry targeted mutations have previously indicated a role for *Gata3* in hair follicle development and skin cell lineage determination. Mice homozygous for germline *Gata3* null mutations die around embryonic day 11 [22,23], precluding a detailed assessment of the functional role of *Gata3* in hair follicle morphogenesis. However, some investigators have rescued mutant skin by transplantation to athymic hosts [24], or else ablated *Gata3* specifically in the epidermis and hair follicles to reveal a crucial role in skin [25]. Since the mouse juvenile alopecia phenotype (patchy hair loss) is distinct from that of these conditionally-targeted mutants (complete baldness)—whatever its molecular basis—we believe that *Gata3*^*jal*^ likely offers a novel mutant allele, compared to the existing set of engineered *Gata3* disruptions. Addition of this viable and phenotypically-unique natural variant to the *Gata3* mutational inventory will surely allow new approaches to the functional analysis of this locus, just as the recent assignment of the spontaneous mouse frizzy (*fr*) and rat “hairless” (*fr*^*CR*^) mutations to the prostasin gene [26] has productively advanced the *in vivo* analysis of *Prss8* function in mammalian skin [27-29].

Haploinsufficiency of human GATA3 (due to loss-of-function mutation of *GATA3*) causes a dominantly-inherited syndrome of hypoparathyroidism, sensorineural deafness, and renal disease (HDR, OMIM #146255) also known as Barakat syndrome. Notably, HDR syndrome does not appear to involve immune-related disorders or alopecia [30,31]. The mouse *Gata3*^*tm1Gsv*^ mutation has been shown to generate deafness in heterozygotes [32-34], and is considered a model for HDR. It would certainly be interesting to investigate parathyroid, cochlear, and renal function in *Gata3*^*jal*^ homozygotes and heterozygotes. In any case, a molecular explanation for the distinct modes of inheritance and phenotypic presentations of juvenile alopecia in mice versus HDR in humans will require discovery of the precise structure of the *Gata3*^*jal*^ allele.

Histological observation of immune cell infiltrates associated with follicular dystrophy in AA [35,36] combined with Petukhova *et al.*’s linkage of genes involved in both innate and acquired immunity (including *IL-2RA*) to AA susceptibility [18] seem to firmly establish AA as an autoimmune disorder. Although *Gata3* is known to play a crucial role in T cell development [22,37], our elimination of *Il2ra* as the basis of the mutant phenotype as well as McElwee *et al.*’s failure to detect any signs of hair follicle inflammation in *jal/jal* mutants [10] suggest that mouse juvenile alopecia does *not* provide an ideal model for AA. However, it remains possible that juvenile alopecia could provide an animal model for at least some forms of focal alopecia which may have their primary defect in the hair follicle and lack an inflammatory component, but which may nonetheless be diagnosed as AA based on similar pathophysiology (*i.e.*, patchy hair loss). Indeed, the future study of mouse juvenile alopecia may be helpful in identifying such a homologous human condition, defining approaches for distinguishing that disorder from AA, and in developing appropriate, specialized treatments.

## Conclusions

The recessive *jal* mutation in mice maps to proximal Chr 2, and has been shown by complementation testing to be a variant allele of the *Gata3* gene. While further study will be needed to discover the molecular defect in *Gata3* that is the basis of the mutant phenotype, this spontaneous mouse variant promises to provide an animal model for some forms of focal alopecia in humans that have their primary defect in the hair follicle and lack an inflammatory component.

## Competing interests

The authors declare that they have no competing interests.

## Authors’ contributions

FR led all aspects of the genome-wide linkage screen, including experimental design, data acquisition and interpretation. AMF led all aspects of the Chr 2 fine-mapping, *Gata3* complementation testing, and sequencing of *Gata3*; including experimental design, data acquisition and interpretation. KMC, LAR and AMF conducted the *Gata3* cDNA analysis. NV-S conducted complementation testing between *jal* and *Il2ra*. EBA, AS, JMH and DVS made substantial contributions to the genome-wide and regional genetic analyses. LAR, KMC and SRH contributed significantly to the SNP marker analysis. TRK conceived of the study, carried out all procedures involving mice, and drafted the manuscript. All authors read, edited, and approved the final manuscript.

## Authors’ information

TRK is a professor in the Department of Biomolecular Sciences at Central Connecticut State University (New Britain, CT). FR was a student in the Master of Arts program in Biomolecular Sciences, and AMF, EBA, AS, NV-S, JMH, LAR, KMC, SRH and DVS were undergraduates majoring in Biomolecular Sciences or Biochemistry at CCSU when they conducted this research.

## Supplementary Material

Additional file 1 **Three-month-old mutants from a (C3H/HeJ-*jal*/J x C57BL/6 J)F**_**1 **_**× C3H/HeJ-*jal*/J backcross display variable expressivity of the juvenile alopecia phenotype.** Click here for file

Additional file 2 **Description of SNP markers referred to in the Ramirez *et al*. (2013) text.** Click here for file

Additional file 3 **Location of SNP markers referred to in the Ramirez *et al*. (2013) text.** Click here for file

Additional file 4 **F**_**1 **_**data from reciprocal crosses in mice tests the juvenile alopecia mutation (*jal*) for X versus autosomal linkage.** Click here for file

Additional file 5 **DNA typing for the *Il2ra***^***tm1Dw***^**or *Il2ra***^***+***^**alleles among the progeny of a complementation cross, *Il2ra***^***tm1Dw***^**/*Il2ra***^***+***^**x *jal/jal.*** Click here for file

Additional file 6 **The recessive *jal *and *Il2ra***^***tm1Dw***^**mutations complement in doubly heterozygous mice.** Click here for file

Additional file 7 **Sequence analysis of *Gata3 *splice junctions in wild-type C3H/HeJ and mutant C3H/HeJ-*jal *cDNA.** Click here for file
