# Supplementary material for: The juvenile alopecia mutation (jal) maps to mouse Chromosome 2, and is an allele of GATA binding protein 3 (Gata3)
Source: BMC Genet. 2013 May 9;14:40. doi: 10.1186/1471-2156-14-40 (PMC3656803; doi:10.1186/1471-2156-14-40)
Supplement: Additional file 1 — Three-month-old mutants from a (C3H/HeJ-jal/J x C57BL/6 J)F1 × C3H/HeJ-jal/J backcross display variable expressivity of the juvenile alopecia phenotype. [file 1471-2156-14-40-S1.pdf]

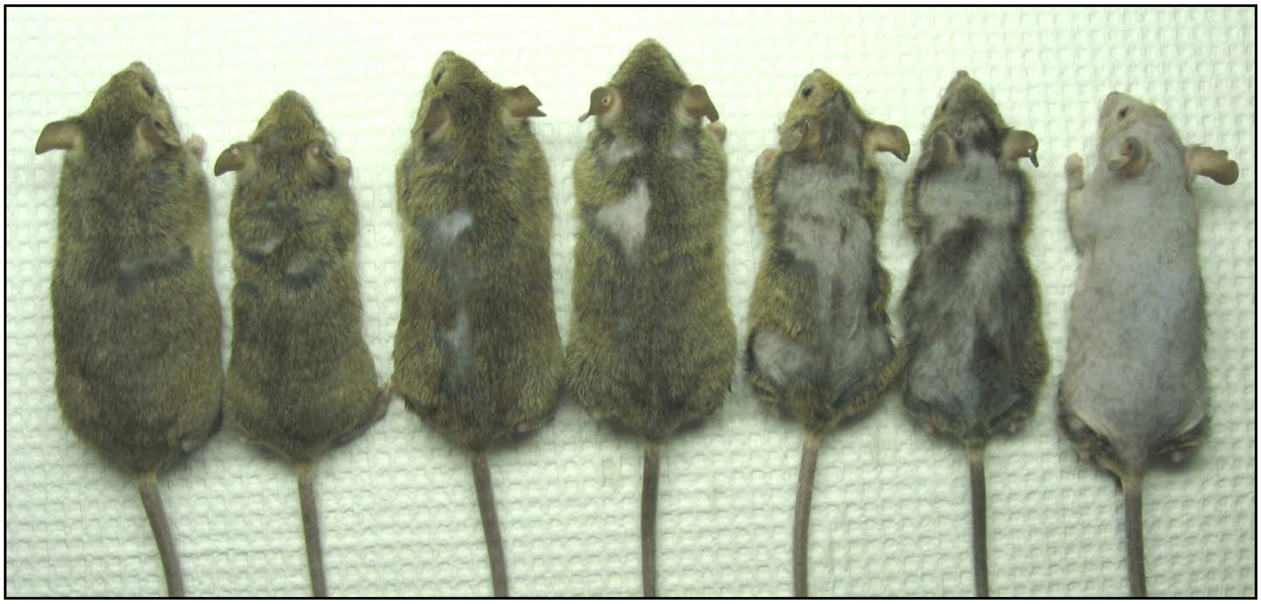

Additional file 1. Three-month-old mutants from a (C3H/HeJ-*jal*/J x C57BL/6J)F_1 x C3H/HeJ-*jal*/J backcross display variable expressivity of the juvenile alopecia phenotype.
