# Supplementary material for: The juvenile alopecia mutation (jal) maps to mouse Chromosome 2, and is an allele of GATA binding protein 3 (Gata3)
Source: BMC Genet. 2013 May 9;14:40. doi: 10.1186/1471-2156-14-40 (PMC3656803; doi:10.1186/1471-2156-14-40)
Supplement: Additional file 2 — Description of SNP markers referred to in the Ramirez et al. (2013) text. [file 1471-2156-14-40-S2.pdf]

Additional file 2. Description of SNP markers referred to in the Ramirez *et al*. (2013) text.

Designation in Ramirez *et al.* 2012	Official Designation	Informal Designation	Forward Primer (5'-3')	Reverse Primer (5'-3')	Amplimer Length	Alleles (A/H=J)	Amplimer Sequence
SNP1	*rs27112885*	SNP O	aatgaaggcaggcagtgaac	agttccttgaagcccaggat	355	G/A	5' aatgaaggca ggcagtgaac<br>TCTCGGCTGA ACTCTTCAAA GAAACAGAAC<br>AACTGTCACT TAGACTCTTA CACCATCTTT<br>CCCATAACTA GTTCATAAAG AGTCAACTGA<br>ATATCAGCAA C**R**ATTTATTT TCAACTCTCT<br>AGCCTTAGGT TTCAACACCT GACCTCAGAT<br>CAATAGACTT GCAAAGGTCT TTCCGTGTGA<br>TCATAAATAT AGTGTAGGCT ATTTTTCTGT<br>GTAATCTCCT CAAGTTGAAG GTAGCTTGGT<br>AGGGCCAAAT TCCACAACCA TCTATTAATT<br>TATTTAGCTC ATTTACAGTT TCTATGAAGG<br>ACAGACACTG TTCTTatcct gggcttcaag<br>gaact 3'
SNP2	*rs27131573, rs27131571*	SNP N	tttgactaagcaggggaacg	ggccagactcaagaatttgc	343	G/C, A/G	5' tttgactaag caggggaacg<br>TGGAGAGAGG CAGATCACTG GAGCTCACTG<br>GCCAACCAGC CTAGCTGAGT CGCCGA**S**CTT<br>CAGGTTTAGC TTGTGAGCCT GACGTAAAAA<br>CTAAGGTAGA GCTCACTCTC TACCTTGAGG<br>AAGATACCTG ATGTTGGCCT CT**R**GTCTCCA<br>CATACATGAC CATGTATACA CTTGCATGTG<br>TAGCTGCACA TACACGTGCA CACACTTACA<br>CAGGCACACA GATGTAACAC ACATGCACAC<br>AAAGATCCAG GGATAGCAAA ACTGTGGACC<br>TAGAACAACT GAGCATGACT TCATGAGTTC<br>TGGgcaaatt cttgagtctg gcc 3'
SNP3	*rs27100936*	SNP J	ggctgatggactaccattgc	cacagcacagcaaagctcat	103	T/C	5' ggctgatgga ctaccattgc<br>GGACTCTATT TGTTGGTGAA AATA**Y**GTGTT<br>CATGTTAGTG CTAGTAATTA CCTCTAGAAA<br>ATAatgagct ttgctgtgct gtg 3'
SNP4	*rs13476354*	SNP I	aaagtctcaacgggcaggat	tccgggcaaattattaactca	384	A/C	5' aaagtctcaa cgggcaggat<br>GCTAAGGTAC CTGCATTTCT TTTCTTACAA<br>AGTATGAGCT GAAAACAATT CTGCTGTCAA<br>AGTTAATCCA CAAAGTGCAC TTGAGACCAT<br>TTTAAAATTT CAACCTGTCT AAACTGCATT<br>TTCAATTGAT AGTGATAGAA TATTATAATA<br>AAAGATGAAA TAAAATATAT GTTACTGTAA<br>TCTCAATCTT AAATTATACA GGATATTAGC<br>ACAGTGCTGA CAGTCAGGAG AGTATAGTAA<br>TATAAAACAG GATGTAGTCA AGAATATTCA<br>AGTGAAGT**M**A AAGTGCATTA ATTACAGAAT<br>TTTATAGAAT CTAACCTGGT GGCATTAAAG<br>TCAATATAAA GGCtgagtta ataatttgcc<br>cgga 3'

Primers listed (in lower case) were used to amplify genomic DNA from strains A/J (abbreviated A), C3H/HeJ (abbreviated H), and C3H/HeJ-*jal*/J (designated J). Amplimers were sequenced by primer extension (SeqWright, Inc.; Houston Texas). Nucleotides that differ between two strains are shown in red, where M = A or C, S = C or G, R = A or G, and Y = C or T. Official designations from dbSNP Build 128. These data accessed through the Mouse Genome Database (MGD) at the Mouse Genome Informatics website, The Jackson Laboratory, Bar Harbor, ME. World Wide Web (URL: http://www.informatics.jax.org; Accessed October, 2012).
