# Supplementary material for: The juvenile alopecia mutation (jal) maps to mouse Chromosome 2, and is an allele of GATA binding protein 3 (Gata3)
Source: BMC Genet. 2013 May 9;14:40. doi: 10.1186/1471-2156-14-40 (PMC3656803; doi:10.1186/1471-2156-14-40)
Supplement: Additional file 3 — Location of SNP markers referred to in the Ramirez et al. (2013) text. [file 1471-2156-14-40-S3.pdf]

Additional file 3. Location of SNP markers referred to in the Ramirez *et al*. (2013) text.

Designation in Ramirez *et al. 2012*	Official Designation	Informal Designation	Position (NCBI Build 37)	5' Flanking Gene	3' Flanking Gene
SNP1	*rs27112885*	SNP O	2: 9587231	*Gm13218*	*Gata3*
SNP2	*rs27131573 & rs27131571*	SNP N	2:10141400, 2:10141486	*Itih5*: Intron	*Itih5*: Intron
SNP3	*rs27100936*	SNP J	2:10314630	*Sfmbt2*: Intron	*Sfmbt2*: Intron
SNP4	*rs13476354*	SNP I	2:13943957	*Ptpla*: Intron	*Ptpla*: Intron

Official designations from dbSNP Build 128. Base-pair positions on mouse Chromosome 2 are from NCBI Build 37. These data accessed through the Mouse Genome Database (MGD) at the Mouse Genome Informatics website, The Jackson Laboratory, Bar Harbor, ME. World Wide Web (URL: http://www.informatics.jax.org; Accessed October, 2012).
