# Supplementary material for: The juvenile alopecia mutation (jal) maps to mouse Chromosome 2, and is an allele of GATA binding protein 3 (Gata3)
Source: BMC Genet. 2013 May 9;14:40. doi: 10.1186/1471-2156-14-40 (PMC3656803; doi:10.1186/1471-2156-14-40)
Supplement: Additional file 4 — F1 data from reciprocal crosses in mice tests the juvenile alopecia mutation (jal) for X versus autosomal linkage. [file 1471-2156-14-40-S4.pdf]

Additional file 4. F_1 data from reciprocal crosses in mice tests the juvenile alopecia mutation (*jal*) for X versus autosomal linkage.

	Gender	Phenotype	Observed Number	Expected Number	χ^2	P value
Cross 1: ♀ C57BL/6J x ♂ *jal/jal*						
	Female	Wild	13	14	0.1429	0.71
	Male	Wild	15	14		
Cross 2: ♀ *jal/jal* x ♂ C57BL/6J						
	Female	Wild	9	9.5	0.0526	0.82
	Male	Wild	10	9.5		

For both crosses, two different breeding pairs were used, and the data shown is the combination of three litters. Phenotypes were scored by visual examination of the coat and whiskers at weaning. The number of males and females recovered in each cross is not significantly different from the 1:1 ratio expected. Cross 1 verifies that *jal* is recessive, since all F_1 offspring were wild type. If *jal* were X-linked, then Cross 2 would have resulted in mutant F_1 males and wild type F_1 females. Since these F_1 males (and females) were, instead, all wild type, we conclude that *jal* must be carried on an autosome.
