# Supplementary material for: The juvenile alopecia mutation (jal) maps to mouse Chromosome 2, and is an allele of GATA binding protein 3 (Gata3)
Source: BMC Genet. 2013 May 9;14:40. doi: 10.1186/1471-2156-14-40 (PMC3656803; doi:10.1186/1471-2156-14-40)
Supplement: Additional file 5 — DNA typing for the Il2ratm1Dwor Il2ra+alleles among the progeny of a complementation cross, Il2ratm1Dw/Il2ra+x jal/jal. [file 1471-2156-14-40-S5.pdf]

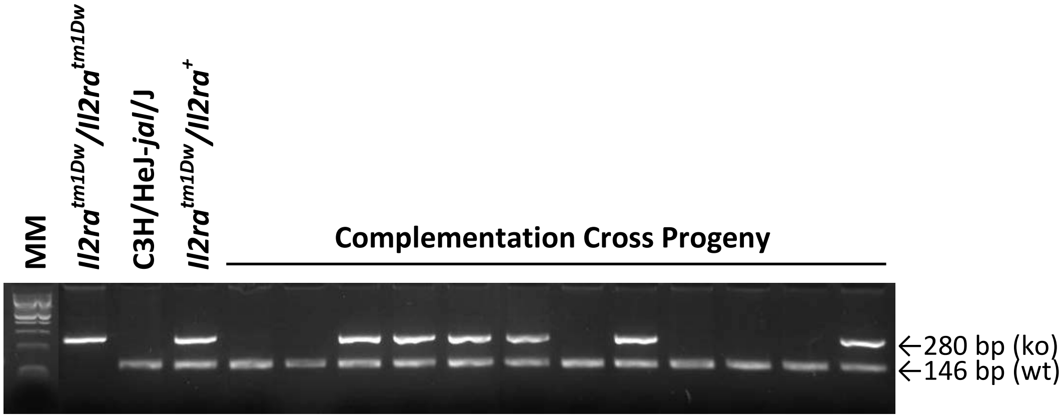


**Additional file 5.** DNA typing for the *Il2ra*$^{tm1Dw}$ or *Il2ra*$^{+}$ alleles among the progeny of a complementation cross, *Il2ra*$^{tm1Dw}$/*Il2ra*$^{+}$ x *jal/jal*. This four-primer PCR assay was recommended by the animal supplier (The Jackson Laboratory, Bar Harbor, ME, USA), and is described in the Methods section.
