# Supplementary material for: The juvenile alopecia mutation (jal) maps to mouse Chromosome 2, and is an allele of GATA binding protein 3 (Gata3)
Source: BMC Genet. 2013 May 9;14:40. doi: 10.1186/1471-2156-14-40 (PMC3656803; doi:10.1186/1471-2156-14-40)
Supplement: Additional file 6 — The recessive jal and Il2ratm1Dwmutations complement in doubly heterozygous mice. [file 1471-2156-14-40-S6.pdf]

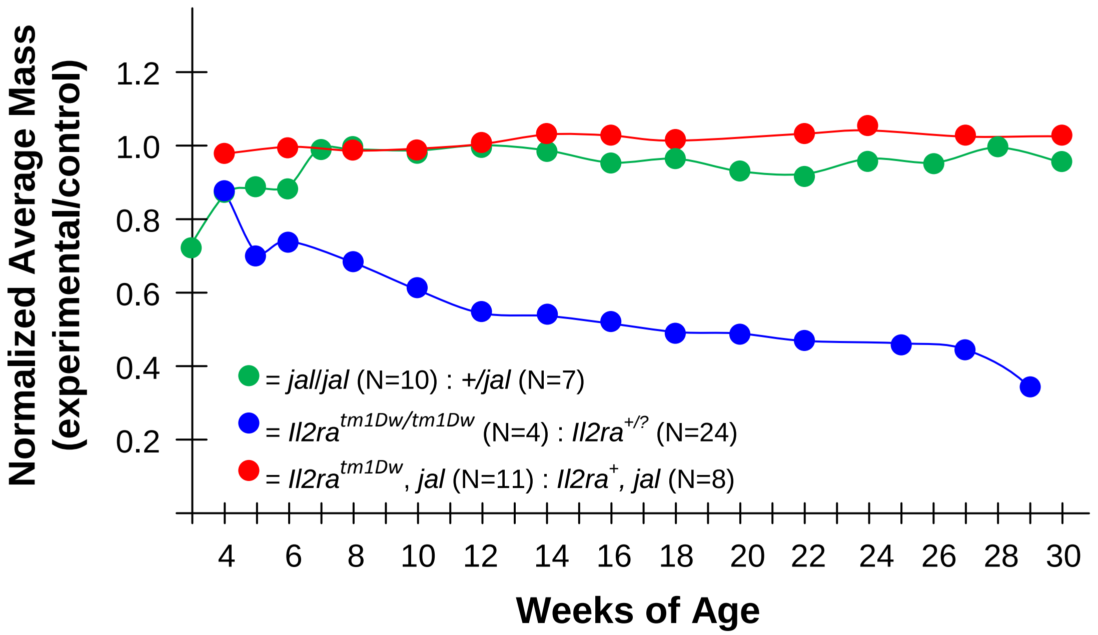

Additional file 6. The recessive *jal* and *Il2ra*^{tm1Dw} mutations complement in doubly heterozygous mice. Three distinct crosses produced "experimental" mice with two recessive mutations (*jal/jal;* or *Il2ra*^{tm1Dw}*, jal;* or *Il2ra*^{tm1Dw}/*Il2ra*^{tm1Dw}) and "control" littermates with at least one wild type allele. All mice were weighed to the nearest 0.5 g every 2-3 weeks. The weights of mice from the same category and gender were averaged, and then the experimental average was divided by the control average. Shown is the average of these normalized values for males and females within each cross at each time point. *Il2ra*^{tm1Dw}/*Il2ra*^{tm1Dw} homozygotes (blue) show a dramatic loss of weight (cachexia) due to an autoimmune syndrome that includes inflammatory bowel disease [19]. Mice homozygous for *jal/jal* (green) are often smaller than control littermates at weaning, but this size difference becomes less apparent with time. The average mass of mice carrying one mutant copy of *jal* and one mutant copy of *Il2ra*^{tm1Dw} (red) is no different from control littermates at any age, suggesting that the *jal* defect does not lie in *Il2ra*. In that case these doubly-heterozygous mice would be wild type dihybrids, genotypically *jal*/+, *Il2ra*^{tm1Dw}/*Il2ra*$^{+}$.
