# Supplementary material for: The juvenile alopecia mutation (jal) maps to mouse Chromosome 2, and is an allele of GATA binding protein 3 (Gata3)
Source: BMC Genet. 2013 May 9;14:40. doi: 10.1186/1471-2156-14-40 (PMC3656803; doi:10.1186/1471-2156-14-40)
Supplement: Additional file 7 — Sequence analysis of Gata3 splice junctions in wild-type C3H/HeJ and mutant C3H/HeJ-jal cDNA. [file 1471-2156-14-40-S7.pdf]

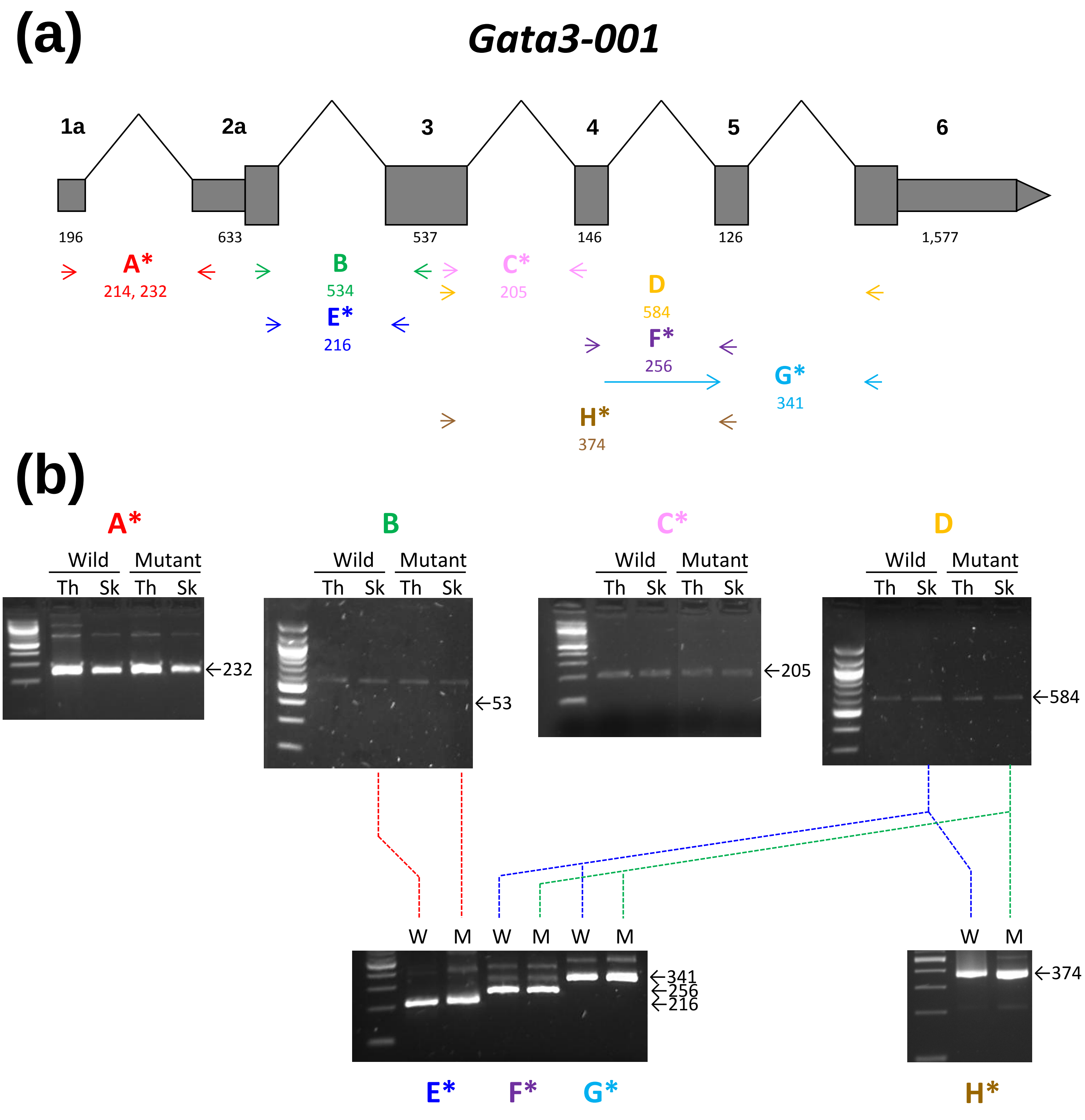

Additional file 7. Sequence analysis of *Gata3* splice junctions in wild-type C3H/HeJ and mutant C3H/HeJ-*jal* cDNA. **(a)** Taller boxes represent coding regions, shorter boxes represent 5' and 3' untranslated regions (UTRs). The length of each exon (in bp) is shown below the corresponding box. The length of each splice-junction-flanking amplimer (A through H) is shown (in bp) between the corresponding primer pair. **(b)** Amplimers A, B, C and D were copied from total RNA isolated from skin (Sk) and thymus (Th) of wild-type (W) C3H/HeJ and mutant (M) C3H/HeJ-*jal*/J mice. Amplimer E was copied from amplimer B; and amplimers F, G, and H were copied from amplimer D. The amplimers marked with an asterisk were sequenced by primer extension analysis (SeqWright, Inc.; Houston, TX), and no differences between wild and mutant transcripts were found. Three *Gata3-201*-specific primer pairs tested failed to amplify products from any skin or thymus, wild type or mutant cDNA sample, even after two rounds of amplification, suggesting that the *Gata3-201* transcript is expressed at a much lower level than the *Gata3-001* transcript, if at all, in thymus and skin. The size standard in the left-most lane on each 3.5 % NuSieve agarose gel is a 100-bp ladder.
